# Comparative neutralizing potencies of antibodies suggest conservation as well as mechanistic differences in human cytomegalovirus entry into epithelial and endothelial cells

**DOI:** 10.1186/s12985-020-01320-2

**Published:** 2020-04-08

**Authors:** Ying Qi, Li He, Xiaohong Cui, Laura Hertel, Daniel C. Freed, Tong-Ming Fu, Lawrence M. Kauvar, Michael A. McVoy, Qiang Ruan

**Affiliations:** 1 Virology laboratory, Shengjing Hospital, China Medical University, Shenyang, People’s Republic of China; 2 Virginia Commonwealth University, Richmond, VA USA; 3 Children’s Hospital Oakland Research Institute, Oakland, CA USA; 4 Merck & Co., West Point, PA USA; 5 University of Texas Health Science Center at Houston, Houston, TX USA; 6 Trellis Bioscience, LLC, Redwood City, CA USA

**Keywords:** Cytomegalovirus, Antibodies, Neutralization, Endothelial, Epithelial, Entry mechanisms

## Abstract

Antibody neutralization of cytomegalovirus (CMV) entry into diverse cell types is a key consideration for development of vaccines and immunotherapeutics. CMV entry into fibroblasts differs significantly from entry into epithelial or endothelial cells: fibroblast entry is mediated by gB and gH/gL/gO, whereas both epithelial and endothelial cell entry require an additional pentameric complex (PC) comprised of gH/gL/UL128/UL130/UL131A. Because PC-specific antibodies in CMV-seropositive human sera do not affect fibroblast entry but potently block entry into epithelial or endothelial cells, substantially higher neutralizing potencies for CMV-positive sera are observed when assayed using epithelial cells as targets than when using fibroblasts. That certain sera exhibit similar discordances between neutralizing potencies measured using epithelial vs. endothelial cells (Gerna G. et al.J Gen Virol, 89:853–865, 2008) suggested that additional mechanistic differences may also exist between epithelial and endothelial cell entry. To further explore this issue, neutralizing potencies using epithelial and endothelial cells were simultaneously determined for eight CMV-positive human sera, CMV-hyperimmune globulin, and a panel of monoclonal or anti-peptide antibodies targeting specific epitopes in gB, gH, gH/gL, or the PC. No significant differences were observed between epithelial and endothelial neutralizing potencies of epitope-specific antibodies, CMV-hyperimmune globulin, or seven of the eight human sera. However, one human serum exhibited a six-fold higher potency for neutralizing entry into epithelial cells vs. endothelial cells. These results suggest that epitopes exist that are important for epithelial entry but are less critical, or perhaps dispensable, for endothelial cell entry. Their existence should be considered when developing monoclonal antibody therapies or subunit vaccines representing limited epitopes.

## Background

Cytomegalovirus (CMV) is a significant cause of birth defects among newborns infected in utero and of morbidity and mortality in transplant and AIDS patients. Therapeutic monoclonal antibodies and prophylactic vaccines targeting humoral responses are in development. Antibodies that neutralize entry of CMV in vitro have been a major focus. However, because CMV entry mechanisms are complex and vary between different cell types, it is uncertain which viral antigens can elicit protective humoral responses in response to vaccination and which can provide efficacious targets for passive immunotherapy.

Initial virion/cell attachment is thought to occur through interactions between cell surface glycosaminoglycans and a dimeric complex of viral glycoproteins M and N (gM/gN) on the virion envelope [1]. Subsequent fusion and entry steps depend on the cell type. In fibroblasts, fusion occurs at the plasma membrane and is mediated by an interplay between the fusogenic glycoprotein B (gB) and a trimeric complex comprised of glycoproteins H, L, and O (gH/gL/gO). In contrast, entry into epithelial, endothelial, and certain myeloid lineage cells involves endocytosis followed by endosomal acidification and, in addition to gB and gH/gL/gO, requires a pentameric complex (PC) comprised of gH/gL plus UL128, UL130, and UL131A [2–5]. Consequently, antibodies to epitopes in gM/gN, gB, gH/gL, and gO can neutralize CMV entry into a variety of cell types, but because the PC is dispensable for fibroblast entry, antibodies to PC-specific epitopes (i.e., involving UL128, UL130, or UL131A) have no neutralizing activity against fibroblast entry [6, 7]. For reasons that remain unclear, PC-specific antibodies often neutralize epithelial or endothelial cell entry with potencies two to three logs higher than those targeting the other complexes [6, 7]. The relative importance of PC-specific antibodies vs. more broadly neutralizing but generally less potent antibodies in preventing CMV-associated disease in immunosuppressed or congenitally infected individuals remains unclear.

Consequently, most current studies focusing on humoral immunity to CMV quantify neutralizing potencies using both fibroblast- and epithelial cell-based in vitro assays. Endothelial cell-based assays have become increasingly less common, presumably due to the assumption that CMV enters epithelial and endothelial cells by similar if not identical mechanisms [3]. Consequently, antibodies are expected to neutralize epithelial and endothelial cell entry with similar potencies. However, in patients undergoing CMV reactivation following solid organ transplantation, differences as high as 16-fold were observed between neutralizing titers measured with epithelial cells vs. endothelial cells, while differences of two-fold or less were observed in pregnant women undergoing primary CMV infections [8]. These results suggest that although the PC is required for efficient CMV entry into both cell types, mechanistic differences may exist between epithelial and endothelial cell entry. Such differences could manifest as discordant sensitivities to antibodies targeting specific epitopes (i.e., antibodies that neutralize entry into one cell type but not the other, or exhibit significant variations in potency). Consequently, significant titer differences in certain antisera could arise if such epitopes dominate the humoral response.

## Main text

In order to determine if qualitative or quantitative discordances exist in the ability of human CMV-positive sera to neutralize CMV entry into epithelial vs. endothelial cells, quantitative green fluorescent protein (GFP)-based neutralizing assays were performed as described previously with minor modifications [9, 10]. The GFP-tagged CMV UxcAp66 [10] was used due to its ability to enter human epithelial and endothelial cells with similar efficiencies (Qi et al., unpublished observations). Human sera were obtained from healthy adults and screened for CMV seropositivity by gB-ELISA as previously described [11]. CMV-positive sera were serially diluted in cell culture medium, mixed with an equal volume of inoculum containing virus and incubated for 1 h at 37 °C, then replicate aliquots were transferred in triplicate to wells of black-walled, clear-bottom 96-well plates containing confluent ARPE-19 epithelial cells (ATCC CRL-2302) or human aortic endothelial cells (HAEC, a gift from Dong Yu). After incubation for 3–7 days, representative images were taken using a Nikon Diaphoto 300 UV microscope and relative fluorescent units of GFP were measured for each well using a Biotek Synergy HT Multi-Mode Microplate Reader. 50% inhibitory concentration (IC_50_) values were determined using Prism 5 (GraphPad Software, Inc.) as the inflection points of four-parameter curves fitted to plots of mean relative fluorescent units (from triplicate wells) vs. Log (antibody concentration) as described previously [12].

**Fig. 1:**
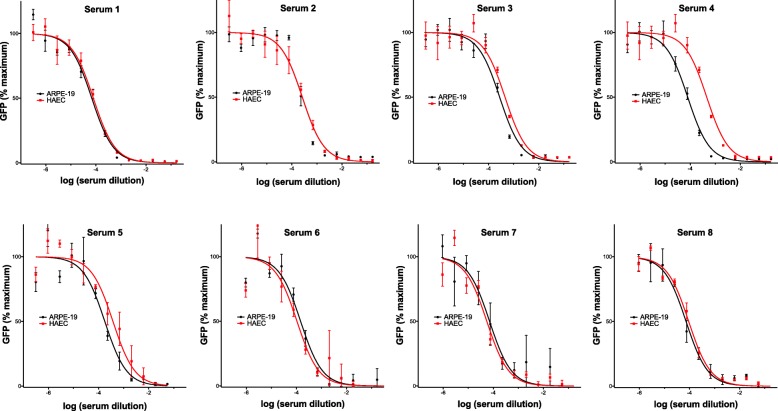
Neutralization of CMV entry into epithelial and endothelial cells by human sera. Neutralizing activities of sera from eight normal CMV-positive human donors were determined using ARPE-19 epithelial cells or HAEC endothelial cells infected with the GFP-tagged CMV strain UxcAp66. GFP values measured seven days post infection were normalized to percent maximum (upper asymptote) and IC_50_ values were determined as the inflection points of four-parameter curves fitted to plots of GFP activity in triplicate wells vs. Log (serum dilution)

Five of eight donor sera tested resulted in epithelial- and endothelial-based neutralizing curves that were closely matched or overlapping (Fig. 1). Two sera (#3 and #5) showed slightly higher potency on epithelial compared to endothelial cells, but the fold differences in IC_50_ values (two-fold or less) were not statistically significant (Table 1, *p* > 0.14, two-tailed t-test). In contrast, the neutralizing curves for donor serum #4 were clearly separated (Fig. 1), with a highly significant 5.75-fold difference in IC_50_ (Table 1, *p* < 0.03, two-tailed t-test).

**Table 1:** Antibody or serum neutralizing activities by cell type

***Species***	***Serum or antibody***	***target***^***a***^	***neutralization IC***_***50***_***(ng/mL)***		***IC***_***50***_***ratio*** ***(endothelial/epithelial)***
			***epithelial***	***endothelial***	
human	serum 1	pan CMV	682	788.3	1.16
	serum 2		2609	2625	1.01
	serum 3		2787	4557	1.64
	serum 4		791	4557	5.75
	serum 5		1769	3742	2.12
	serum6		1467	1052	0.72
	serum 7		710	552.2	0.78
	serum 8		725	924.8	1.28
	HIG^***d***^		105	70.0	0.67
	TRL345	gB	91.0	95.4	1.05
	TRL310	PC	0.286	0.318	1.11
	2–25		0.525	0.374	0.71
rabbit	57.4		22.9	19.7	0.86
	276.1		34.8	24.7	0.71
	α-UL130^b^	PC (UL130)	1429^***c***^	685^***c***^	0.48
	α-UL131^b^	PC (UL131A)	62.6^***c***^	87.9^***c***^	1.40
	15.1	gH	121	116	0.96
	58.5		209	220	1.05
	223.4		156	240	1.54
	347.3		391	292	0.75
	70.7		36.5	29.5	0.81
	124.4	gH/gL	67.5	49.1	0.73
	270.7		36.7	19.2	0.52
	316.2		49.8	34.2	0.69
	324.4		79.4	50.8	0.64

^a^see Main Text for references

^b^polyclonal rabbit anti-peptide sera

^c^serum values are based on an assumed serum IgG concentration of 10 mg/mL

^d^CytoGam® (purified pooled polyclonal human IgG)

These results suggested that serum from donor #4 contains antibodies targeting unique epitopes critical for entry into epithelial cells, but less important or perhaps dispensable for entry into endothelial cells. We therefore used the same assay to ascertain both epithelial- and endothelial-based neutralizing potencies of a panel of epitope-specific monoclonal antibodies (mAbs) and anti-peptide rabbit antisera, as well as hyperimmune globulin (HIG), a commercial preparation of human IgG purified from CMV-positive donors (CytoGam®, CSL Behring, King of Prussia, PA) (Table 1). The panel included: (i) eleven CMV-neutralizing mAbs targeting gH, gH/gL, or the PC that were isolated from a rabbit immunized with an experimental whole virus vaccine based on CMV strain AD169 [7, 10, 13]; (ii) two polyclonal rabbit antisera that were raised against 17–20 amino acid long synthetic peptides representing sequences within UL130 or UL131A [5, 12]; (iii) two human mAbs, 2–25 and TRL310, that recognize the PC [13, 14], and (iv)) one human mAb, TRL345, that recognizes the AD-2 epitope in gB [14, 15] (Table 1).

**Fig. 2:**
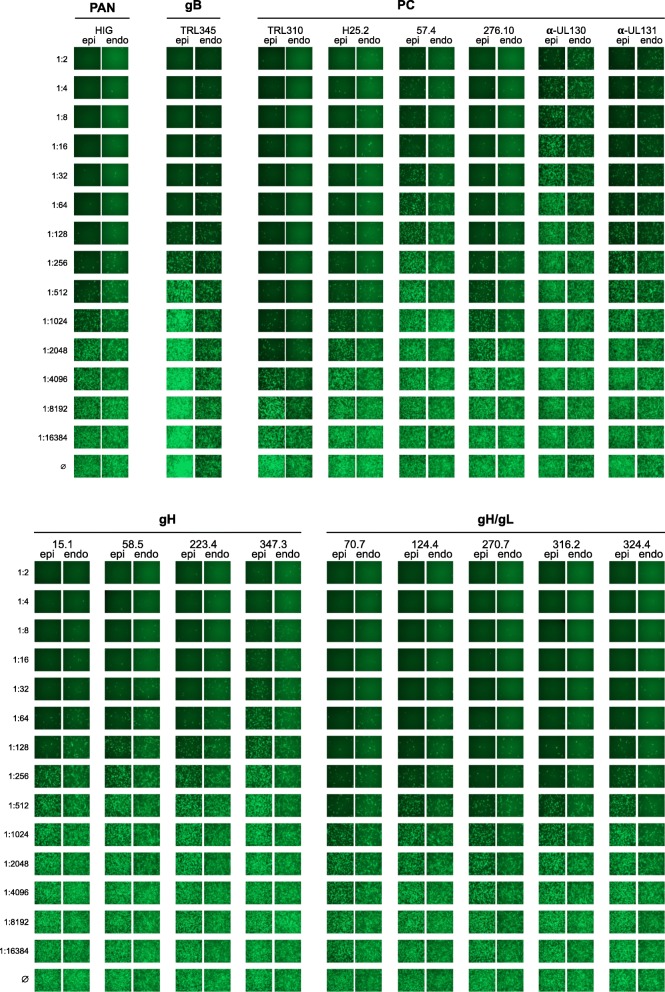
Neutralization of CMV entry into epithelial and endothelial cells by HIG and epitope-specific antibodies. (A) Neutralizing activities of antibodies were determined using ARPE-19 epithelial cells (epi) or HAEC endothelial cells (endo) infected with the GFP-tagged CMV strain UxcAp66. Wells were photographed with an inverted UV microscope four (ARPE-19) or six (HAEC) days after infection. Sample dilutions are indicated on the left; ∅, virus incubated with media (no antibody)

**Fig. 3:**
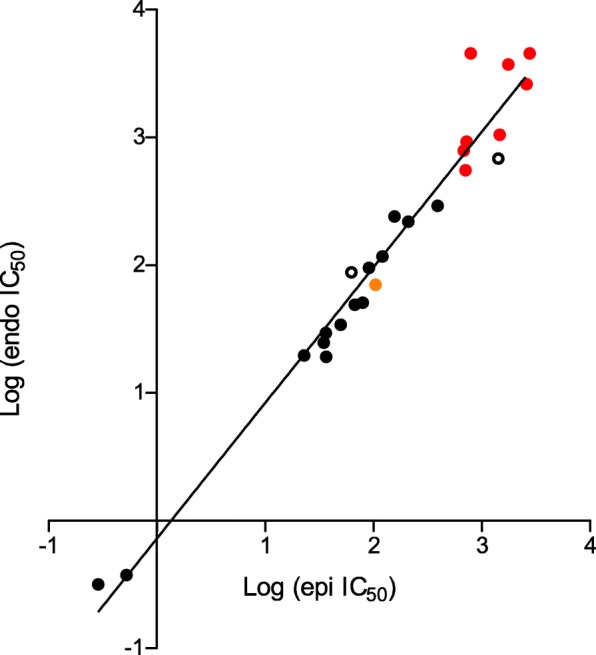
Epithelial and endothelial neutralizing titers exhibit a strong correlation. Log transformed IC_50_ values for endothelial vs. epithelial neutralizing activity were plotted for each of the antibodies and sera listed in Table 1 and analyzed by logistic regression using Prism 5 software (GraphPad Software, Inc.). Dot colors correspond to human and rabbit mAbs (black), rabbit anti-peptide sera (white), HIG (orange), and human sera (red). Serum IC_50_ values are expressed in units of ng of IgG/mL, assuming an IgG concentration of 10 mg/mL

Qualitative discordances were not observed and IC_50_ values for epithelial and endothelial neutralization differed by less than two-fold (Fig. 2 and Table 1). When logistic regression was used to compare the neutralizing IC_50_ values of all antibodies and sera tested, a high degree of correlation (r = 0.98) was observed between the two cell types, despite inclusion of the outlier serum #4 (Fig. 3).

Prior to the discovery that CMV enters epithelial and endothelial cells via mechanism(s) that are distinct from those used to enter fibroblasts, virtually all neutralizing assays utilized fibroblasts. The finding that the PC is required for epithelial/endothelial cell entry but not for fibroblast entry prompted studies to determine if neutralizing antibody potencies are significantly influenced by the cell type. Sera from naturally infected human subjects were found to be substantially more potent in neutralizing entry into endothelial and epithelial cells than into fibroblasts [8, 16–18], and through adsorption studies, the additional epithelial-specific component of neutralizing activity was attributed to antibodies specific to the PC [19, 20]. Importantly, the observation that candidate vaccines with suboptimal efficacy were poorly immunogenic with respect to inducing epithelial-specific neutralizing responses suggested that improved efficacy could potentially be achieved by increasing epithelial cell-specific neutralizing responses [16].

Measurement of both epithelial- and fibroblast-specific neutralizing activities has since become standard when characterizing immunogenicity of candidate CMV vaccines or immunotherapeutics. However, just as exclusive reliance on fibroblasts concealed and delayed the discovery of epithelial-specific neutralizing antibodies, and inadequately characterized the immunogenicity of vaccine candidates, an analogous risk may exist in using only epithelial cells to measure neutralizing activities. Unappreciated and potentially subtle differences between epithelial and endothelial cell entry may thus result in antibodies to certain epitopes exhibiting greater potency against entry into one cell type vs. the other. While such differences may not be readily observable in the context of polyclonal responses to a broad range of neutralizing epitopes, more focused responses, such as those induced by subunit vaccines, could potentially induce neutralizing responses that are robust when measured using epithelial cells but modest when measured using endothelial cells, or vice versa.

That such differences may exist between epithelial and endothelial cell entry was first suggested by data reported by Gerna et al. in which semi-quantitative assays (with approximately two-fold accuracy) indicated differences between epithelial and endothelial neutralizing potencies of two- to eight-fold (and for one serum 16-fold) in serial sera from five patients experiencing CMV reactivation after solid-organ transplantation [8]. While these results were noted, their implications regarding potential mechanistic differences in epithelial vs. endothelial cell entry were not discussed. To confirm and extend these reported observations, we examined the epithelial and endothelial neutralizing potencies of sera from healthy, naturally infected human subjects using highly comparable, objective, and quantitative methods. While the neutralization curves of six of the eight human sera were nearly superimposable, one sera exhibited ~two-fold higher epithelial vs. endothelial IC_50_, while a second serum exhibited a statistically significant six-fold difference.

Efforts to identify specific epitopes that might underlie such potency differences did not reveal significant discordances within a panel of epitope-specific antibodies, and overall, logistic regression analysis showed a high correlation between the two neutralizing activities. However, this antibody panel likely represents only a limited subset of all epitopes that mediate neutralization of epithelial and/or endothelial entry. While antibodies in the panel may recognize up to six unique PC-specific epitopes, only one gB antibody was included, and although seven additional mAbs target epitopes in gH or gH/gL, binding interference assays suggest that these seven antibodies may target only two unique epitopes [13]. Moreover, the panel lacked antibodies representing epitopes in other CMV entry mediators, such as gM, gN, and gO, that are known targets of neutralizing antibodies [21, 22].

Nonetheless, our finding that one in eight human sera exhibits a significant difference in serum neutralizing potency confirms and extends the results reported by Gerna et al. [8], and together these findings suggest that antibodies with differential neutralizing activities exist, and that in certain subjects the epitopes targeted by such antibodies can comprise a significant component of the neutralizing response repertoire. Additional studies will be needed to determine the prevalence of differentially neutralizing activities in different subject populations, to identify the relevant viral proteins through adsorption experiments, and to define the specific epitopes that mediate differential neutralization through the analysis of a more diverse panel of antibodies.

These findings support the use of a standard cell type such as ARPE-19 cells to capture neutralizing activities against PC-mediated CMV entry and suggest that ARPE-19 cell-based assays may be appropriate and sufficient for most natural history studies. Epitopes may nevertheless exist that are not important for endothelial entry, but are involved or perhaps critical for epithelial cell entry. The potential existence of such epitopes should be considered when developing monoclonal antibody therapies or subunit vaccines representing limited epitope repertoires.

## Data Availability

All data generated or analyzed during this study are included in this published article.

## Abbreviations

CMV: Cytomegalovirus
GFP: Green fluorescent protein
gB: Glycoprotein B
gH: Glycoprotein H
gL: Glycoprotein L
gM: Glycoprotein M
gN: Glycoprotein N
gO: Glycoprotein O
HIG: Hyperimmuneglobulin
IC_50_: 50% inhibitory concentration
mAbs: Monoclonal antibodies
PC: Pentameric complex
